# Socioeconomic factors in relation to dental caries among children aged 5–14 years: a cross-national comparative study using secondary data analyses

**DOI:** 10.1186/s12903-025-06959-3

**Published:** 2025-10-21

**Authors:** Yu-An Yang, Yao-Hui Huang, Yi-Hao Weng, Ya-Wen Chiu

**Affiliations:** 1 Master Program in Global Health and Development, Ph.D Program in Global Health and Health Security, College of Public Health, Taipei Medical University, Taipei, Taiwan; 2 School of Dentistry, National Defense Medical Center, Taipei, Taiwan; 3 Department of Pediatrics, Chang Gung Memorial Hospital, Chang Gung University College of Medicine, Taipei, Taiwan; 4 Institute of Population Health Sciences, National Health Research Institutes, Miaoli, Taiwan

**Keywords:** Dental caries, Water fluoridation, Children, Deciduous teeth, Permanent teeth

## Abstract

**Background:**

Dental caries is a preventable non-communicable disease. Untreated caries in deciduous teeth may contribute to the development of caries in permanent teeth. Nevertheless, limited research has focused specifically on the risk factors in children. The current study examined the association between dental caries and socioeconomic risk factors among children aged 5–14 years across countries worldwide.

**Methods:**

An ecological survey was conducted using nation-based, publicly available online databases from six reputable organizations: the World Health Organization, the Food and Agriculture Organization of the United Nations, the World Bank, the British Fluoridation Society, the United Nations Development Programme, and the Global Burden of Disease Project. Data were collected from these sources between 2014 and 2017. Independent variables included density of dental personnel, parental education, family income, water fluoridation, and sugar consumption. The dependent variables were the prevalence of caries in deciduous and permanent teeth. Data were analyzed using descriptive statistics, univariate analysis, and multinomial logistic regression.

**Results:**

After eliminating countries without complete information, this study enrolled 120 countries with complete data for both dependent and independent variables. Univariate analysis revealed significant differences by parental education, income level, and water fluoridation. We further conducted multivariate logistic regression analysis, indicating that countries with low fluoridation (< 50%) had significantly higher odds of caries in permanent teeth (OR: 13.23; 95% CI: 1.22–143.53; *p* = 0.03); shorter years of parental schooling was associated with lower prevalence of caries in permanent teeth (OR: 0.12; 95% CI: 0.03–0.47; *p* = 0.002); and middle-income countries showed increased risk in both deciduous teeth (OR: 3.44; 95% CI: 1.26–9.43; *p* = 0.02) and permanent teeth (OR: 6.93; 95% CI: 1.75–27.38; *p* = 0.01) than high-income countries. Sugar consumption and density of dental personnel were not significantly associated.

**Conclusion:**

This ecological study provides valuable insights into the global patterns of dental caries in children aged 5–14 years and their associations with selected socioeconomic indicators. Our results reveal significant correlations of dental caries with income level, water fluoridation coverage, and parental education. However, these associations should be interpreted with caution due to the ecological nature of the data and several important limitations.

## Introduction

Although dental caries is preventable, its prevalence remains high in low-income countries, largely due to persistent social and economic disparities. For example, children from low-income families often have limited access to dental care [1]. In some countries, up to 50% of preschool children have experienced dental caries [2]. Dental caries and associated toothache negatively impact quality of life, causing difficulties in eating, sleeping, and smiling [3]. Children aged 5–14 years undergo the transition from primary (deciduous) to permanent dentition, during which they typically have both tooth types simultaneously. Dental health during this period can influence oral health outcomes throughout adulthood. However, children in this age group are at high risk of dental caries due to increased exposure and unhealthy behaviors. Understanding the factors influencing caries in children aged 5–14 years is therefore essential.

Socioeconomic factors – such as family income, parental education, water fluoridation policy, availability of dental personnel, and sugar consumption – have been associated with dental caries in both deciduous and permanent teeth [4–9]. Water fluoridation is a proven effective method for preventing dental caries; however, concerns remain regarding its potential side effects. Moreover, limited evidence exists on the relationship between socio-behavioral factors and dental caries among children aged 5–14 years from a global, nation-based perspective. Therefore, this study aimed to examine the correlations between dental caries in both deciduous and permanent teeth and five key variables: water fluoridation coverage, mean years of schooling, income level, sugar consumption, and dental personnel density, among children aged 5–14 years in countries worldwide. We hypothesized that these factors contribute to the risk of dental caries. To address this, we utilized six secondary databases to explore the association between dental caries and socioeconomic factors from a global perspective.

## Method

### Study design and study participants

An ecological survey was conducted using six nation-based, publicly available online databases: the World Health Organization, the United Nations Development Programme, the World Bank, the British Fluoridation Society, the Food and Agriculture Organization of the United Nations, and the Global Burden of Disease Project (Table 1). The inclusion criteria comprised data on children aged 5–14 years worldwide, while countries lacking complete data for both dependent and independent variables were excluded. Data were collected from these databases between 2014 and 2017.

**Table 1:** Source of nation-based databases

information	source
Prevalence of dental caries	Global Burden of Disease Project
Parental education	United Nations Development Programme
Family income	World Bank
Water fluoridation policy	British Fluoridation Society
Sugar consumption	Food and Agriculture Organization of the United Nations

### Data collection measures

A Dependent variable: dental caries
  Data on the prevalence of dental caries in both deciduous and permanent teeth were obtained from the Global Burden of Disease Project for 194 countries [10]. Dental caries was defined as the presence of at least one affected tooth.
B Independent variables: socio-economic factors
  Socioeconomic factors – including water fluoridation coverage, sugar consumption, dental personnel density, parental education, and family income – were obtained from nation-based databases as described below.
  1. Water fluoridation
    Data on water fluoridation policy coverage were obtained from the British Fluoridation Society and expressed as the percentage of the population receiving optimally fluoridated water [11].
  2. Sugar consumption
    Sugar consumption data were obtained from the Food and Agriculture Organization of the United Nations. Sugar consumption was defined as the total quantity of sugar and sweeteners available for human consumption, calculated as the sum of domestic production and imports, adjusted for changes in stock levels. The data were expressed in kilograms per capita per year [12].
  3. Dentistry personnel
    Data on dental personnel were obtained from the World Health Organization. High density of dentistry personnel was defined as more than 3.84 dentistry personnel per 1000 population [13].
  4. Parental education
    Parental education data were obtained from the United Nations Development Programme. Mean years of schooling among people aged over 25 years were used as the measure of parental education level [14].
  5. Family income
    Data on income level were obtained from the World Bank. Countries were classified as high-, middle-, or low-income according to the World Bank’s Gross National Income (GNI) per capita classification [15] (Table 2).

**Table 2:** *Classification of* dental caries and socioeconomic factors

Dental caries	deciduous teeth	permanent teeth	Cut point
Low	≤ 0.27	≤ 0.26	Global means
High	> 0 0.27	> 0.26	
**socioeconomic factors**			
**Dentistry personnel**	Per 1,000 population, year		
Low	≤ 3.84		Global mean
High	> 3.84		
**Parental schooling year**	Years, per capita		
Short	≤ 9.2		Global mean
Long	> 9.2		
**Income level**	Gross National Income (GNI) per capita in US$		
Low	≤ 1,025		World Bank
Middle	1,026 − 12,375		
High	> 12,375		
**Water fluoridation**	Coverage [%]		50% coverage
Low	0–50%		
High	51–100%		
**Sugar consumption**	kg per capita, year		Global mean
Low	≤ 42.10		
High	> 42.10		

*GNI* Gross National Income

### Data analysis

Data of dependent and independent variables were dichotomized into two groups except income level for convenient comparison (Table 2). For water fluoridation policy, high coverage of water fluoridation was defined as fluoridation more than 50%. Dental caries, mean years of parental schooling, sugar consumption, and dentistry personnel countries were divided into two groups according to their mean the followings: dental caries in deciduous teeth (mean = 0.27), dental caries in permanent teeth (mean = 0.26), mean years of schooling (mean = 9.2years), sugar consumption (mean = 42.10 kg per capita) and dentistry personnel (mean = 3.84 per 1,000 population).

### Ethical consideration

This ecological study was conducted as a secondary analysis using data obtained entirely from publicly available online sources; therefore, ethical approval was not required.

#### Statistical analyses

All statistical analyses were performed using a commercially available program (SPSS^®^ version 18.0 for Windows; Chicago, IL, USA). Descriptive statistics were used to describe the mean, standard deviation, and frequency of the subjects recruited in the study. Analysis of variance (ANOVA) was used to examine the differences among groups. Multinomial logistic regression was used to determine the risk among socio-behavioral factors. Odds ratio (OR) with 95% confidence interval (CI) was expressed after adjusting for the control variables. Statistical significance was set at *p* < 0.05.

## Results

### Dental caries globally

The worldwide prevalence of caries in deciduous and permanent teeth is shown in Table 3. For deciduous teeth, top ten highest and lowest prevalence of dental caries are Suriname, Qatar, Lithuania, Estonia, Oman, Belarus, South Korea, United Arab Emirates, Georgia, and Latvia (from 0.34 to 0.38) and Nigeria, Switzerland, New Zealand, Trinidad and Tobago, Norway, Tanzania, Canada, Italy, United Kingdom and Myanmar (from 0.10 to 0.19). As for permanent teeth, the prevalence of dental caries is higher in Ecuador, Bolivia, Albania, Bulgaria, Bosnia and Herzegovina, Peru, Montenegro, North Macedonia, and Cote d’Ivoire (from 0.38 to 0.41) and lower in Nigeria, Ghana, Mauritania, New Zealand, Equatorial Guinea, Cameroon, Cape Verde, Australia, and Kenya (from 0.07 to 0.12).

**Table 3:** Information of dental caries and socioeconomic factors in 194 countries from 2014–2017

Prevalence of dental caries	Mean	SD	Minimum	Maximum
Deciduous teeth	0.27	0.05	0.09	0.38
Permanent teeth	0.26	0.08	0.07	0.41
Socio-economic factor	**Mean**	**SD**	**Minimum**	**Maximum**
Water fluoridation	0.05	0.17	0	1.00
Parental schooling year	8.48	3.16	2	14
Gross National Income	14192.96	18,949	289	106,264
Sugar Consumption	37.96	24.31	4	162
Dentistry Personnel	3.89	3.51	0	16

Sugar Consumption: kg/per capita, year

Dentistry Personnel: per 1,000 population, year

After eliminating countries without complete information, the study analyzed 120 countries with complete data for both dependent and independent variables. Among the enrolled 120 countries, the average prevalence of dental caries in both deciduous and permanent dentition was 0.27, aligning closely with global estimates. Among these 120 countries, top ten highest and lowest prevalence of dental caries in deciduous teeth are Suriname, Lithuania, Estonia, Oman, Belarus, South Korea, Georgia, Latvia, Saudi Arabia, Cyprus (from 0.33 to 0.38) and Switzerland, New Zealand, Trinidad and Tobago, Norway, Canada, Italy, Myanmar, United States, Zimbabwe, and Germany (from 0.14 to 0.20). Furthermore, dental caries in permanent teeth is more common in Ecuador, Bolivia, Albania, Bulgaria, Peru, Montenegro, Cote d’Ivoire, Georgia, Poland, Switzerland (from 0.38 to 0.41) and less common in New Zealand, Cameroon, Cape Verde, Australia, Kenya, Japan, Senegal, The Gambia, Gabon, and Benin (from 0.12 to 0.14).

### Correlation of dental caries with socio-economic factors

The classification of dental caries and socio-economic factors is also shown in Table 3. Among 24 countries with water fluoridation, only 14 countries were enrolled into this study, including Malaysia (75.5%), United State (74%), Irish (73%), Israel (70%), Australia (70%), Chile (65%), New Zealand (61%), Canada (44%), Brazil (41%), Fiji (36%), Panama (18%), South Korea (6%), Serbia (3%), and Peru (2%).

First, we used univariate analysis to evaluate the correlation of dental caries with socio-economic factors (Table 4). There was a significant correlation of dental caries in deciduous teeth with income level and water fluoridation. Dental caries of deciduous teeth was more common in countries with middle income (*p* = 0.01) and low water fluoridation (*p* = 0.02). In addition, there was a significant correlation of dental caries in permanent teeth with parental schooling year (*p* = 0.02), income level (*p* = 0.001) and water fluoridation (*p* = 0.004). Dental caries of permanent teeth was more common in countries with longer average years of parental education, middle income and low water fluoridation.

**Table 4:** **Correlation of dental caries with socio-economic factors** for children aged 5–14 in 120 countries **by univariate analysis**

Socio-economic factors	*N* (%)	Deciduous teeth			Permanent teeth		
		Mean	SD	*p* value	Mean	SD	*p* value
**Dentistry personnel** Low				0.38			0.60
	**70 (58.3)**	0.27	0.04		0.27	0.08	
High	**50 (41.7)**	0.27	0.06		0.28	0.08	
**Parental schooling year** Short				0.33			0.02*
	**56 (46.7)**	0.27	0.04		0.25	0.08	
Long	**64 (53.3)**	0.28	0.06		0.29	0.08	
**Income level** Low				0.01*			0.001*
	**10 (8.3)**	0.25	0.04		0.20	0.08	
Middle	**68 (56.7)**	0.28	0.04		0.29	0.07	
High	**42 (35.0)**	0.26	0.06		0.26	0.07	
**Water fluoridation** Low				0.02*			0.004*
	**113 (94.2)**	0.27	0.05		0.28	0.08	
High	**7 (5.8)**	0.24	0.06		0.19	0.06	
**Sugar consumption** Low				0.11			0.51
	**67 (55.8)**	0.28	0.04		0.28	0.09	
High	**53 (44.2)**	0.26	0.05		0.27	0.07	

*SD* Standard deviation. *Statistical significance

Table 5 shows the risk assessment of dental caries by multivariate logistic regression analysis. Countries with middle income were at higher risk of dental caries in decidual teeth than those with high income (OR = 3.4; *p* = 0.02). As for permanent teeth, the highest primary risk factor for dental caries was low water fluoridation coverage (OR = 13.2; *p* = 0.03), followed by longer average years of parental education (OR = 8.3; *p* = 0.002), and middle income (OR = 6.9; *p* = 0.01).

**Table 5:** Risk assessment of dental caries for children aged 5–14 by multivariate logistic regression analysis

Socio-economic factors	Deciduous teeth						Permanent teeth			
	OR (95% CI)		*p* value				OR (95% CI)			*p* value
**Parental schooling year** Short										
	0.45 (0.17–1.19)		0.11				0.12 (0.03–0.47)			0.002*
Long	reference						reference			
**Income level** Low										
	0.80 (0.12–5.42)			0.82			1.31 (0.16–10.90)			0.81
Middle	3.44 (1.26–9.43)			0.02*			6.93 (1.75–27.38)			0.01*
High	reference						reference			
**Water fluoridation** Low										
	0.85 (0.15–4.86)			0.86			13.23 (1.22-143.53)			0.03*
High	reference						reference			

*OR* Odds ratio. *Statistical significance

## Discussion

This study investigated the associations between socio-economic factors and dental caries using country-level data from a global sample of 120 nations. Our results indicated that income level, water fluoridation, and years of schooling were significantly associated with the risk of dental caries. In contrast, no significant correlation was found between dental caries and the number of dental personnel or sugar consumption.

In our study, countries classified as middle-income showed a significantly higher risk for dental caries in both dentitions compared to low-income countries. Middle-income countries may have greater access to unhealthy snacks, increasing the likelihood of dental caries. For example, a study conducted in Iran showed that higher Gross Domestic Product (GDP) would have lower DMFT (decay, missing and filled teeth) in deciduous teeth and permanent teeth [16]. This may be possibly due to dietary differences. Children in middle-income countries tend to consume more processed foods compared to those in low-income countries, where limited purchasing power reduces access to cariogenic foods. Additionally, middle-income countries often allocate fewer resources to preventive policies, such as water fluoridation, salt fluoridation, and fluoride toothpaste programs, compared to high-income countries [17]. Overall, middle income countries might have higher risk to have dental caries as a result of less preventive policy but similar possibilities to access unhealthy snacks.

In our study, countries with below 50% coverage of water fluoridation showed higher risk of dental caries in permanent teeth than the countries with over 50% coverage. Although there were only 6 countries in the study with over 50% coverage of water fluoridation, the results showed significant difference still, which mean it is strong enough to prove the effect of water fluoridation. For deciduous teeth, the result shows differences between the countries with high and low water fluoridation coverage in the univariate analysis. But when we put water fluoridation, income level and mean years of schooling together to look at the risk, compare to the low coverage group, high coverage group did not show significant difference than low coverage group. The findings were similar with previous reports showing water fluoridation a little effect on the prevention of dental caries for children [18–21]. To promote water fluoridation, some people might concern about the syndrome of dental fluorosis, there is another study conducted in 2015 about the impact of dental fluorosis and its influence on children’s life, the majority of children did not perceive fluorosis spots, and the study also demonstrated fluorosis did not affect their quality of life [22]. Thus, water fluoridation should be a cost-effective and harmless strategy to prevent dental caries [18–23].

Our study found that countries with short mean years of parental schooling were at lower risk of dental caries at permanent teeth. Parents with higher education level might be assumed to have better understanding of keeping themselves healthy, and realize the importance of health. However, our results show discrepant findings. We proposed two possible reasons. First, our study did not assess oral health literacy directly although literacy in oral health is more important than educational level. Children of mothers with high oral health knowledge and positive oral health attitudes had lower prevalence of dental caries than those with poor oral health knowledge and attitudes [24]. Therefore, education for accurate knowledge in dental caries should be delivered well, like flossing is the most efficient way to get rid of dental plague and the cleaning technique taught how to brush our teeth properly. Nevertheless, these individual indicators did not be counted into this research. Second, detection bias may inflate prevalence in countries with higher education levels. Parents with more education may seek dental care more often, increasing detection and treatment rates. Thus, higher reported prevalence may reflect better diagnosis rather than higher untreated diseases. For example, it’s possible that parents with high education more often take their children to dental clinics, which leading to increased diagnosis and documentation of dental caries. As a result, the prevalence appears higher, not necessarily because children have more untreated decay, but because more cases are being identified and recorded.

Although sugar consumption is a known risk factor for dental caries [25, 26], our study did not find significant differences between countries with low and high sugar consumption. This is likely because we used average national sugar consumption data, which does not reflect sugar intake specifically among children aged 5–14 years. Due to the limitations of the dataset, we used the average sugar consumption across the entire population of each country, rather than specific data for the 5–14 age group. Thus, our study could not provide sufficient evidence in the correlation of sugar consumption with dental caries.

In our investigation, we did not find a significant relationship between dental caries and dentistry personnel in both deciduous and permanent teeth. A similar result stated that the adequacy of health workers is not directly to the prevalence of active dental caries [5]. In contrast, a study conducted in 2017 demonstrated a significantly negative association between the dentistry personnel and untreated dental caries among children from low-income families in Head Start programs in Northeast Ohio [6]. The reason of inconsistent findings could be that many factors contribute to the prevalence of dental caries. Dentistry personnel can only present whether there is enough dentist to treat dental caries but it cannot represent how the prevention works in this country. In other words, adequate health workers could not represent that the prevalence of dental caries would be low here.

There are some limitations to this study. First, the ecological nature of the study precluded inferences at the individual level (ecological fallacy). Observed associations may not directly translate to individual risk patterns. In addition, we have the problems of data aggregation and missingness. Only countries with complete data for all variables were included, reducing the final sample size and potentially introducing selection bias. Second, our study faced limitations of non-specific exposure metrics, including sugar consumption, dental personnel density, parental education, and water fluoridation. Sugar consumption was reported at the national per capita level, without age-specific data for children, thus limiting relevance to pediatric dental outcomes. Density of dental personnel did not reflect accessibility, affordability, or preventive orientation of dental services. Also, detection bias could happen as countries with higher parental education levels and better access to dental care may have more frequent caries detection and treatment, leading to inflated prevalence figures due to reporting rather than true disease burden. For the water fluoridation, the number of countries with high fluoridation coverage in the analysis was small, which may limit the generalizability of conclusions.

Lastly, the issue of unmeasured confounding should be considered. Important individual-level and behavioral factors, such as frequency of tooth brushing, use of fluoride toothpaste, access to preventive programs, and cultural norms around oral hygiene, were not captured due to the study design.

As for the strength of this study, we conducted a nation-based comparison to identify the potential risk factors of dental caries. Data were collected from 194 countries for 4 years of observation period, and analyze 120 countries among them which got complete data for both dependent and independent variables. Our ecological study could deliver an overview to understand this issue at a global level. The extensive global scope allows for broad generalizations and adds value to the global understanding of pediatric dental caries. Second, the topic of our study is timely and relevant since we investigated the socioeconomic determinants of a preventable public health issue. Third, we used reputable data sources from trusted organizations (e.g., WHO, UNDP, World Bank, FAO), which enhancing the study’s credibility. Fourth, the use of both univariate and multivariate analyses supports the reliability of the findings. Based on our findings, future research could explore cross-country differences in greater depth through primary data collection. This approach would allow for more precise results and tailored recommendations, addressing the specific needs of different countries and age groups using real-time data.

## Conclusion

This ecological study offers valuable insight into the global patterns of dental caries in children aged 5–14 and their associations with selected socioeconomic indicators. Drawing on data from 120 countries, the analysis highlights significant relationships between dental caries prevalence and factors such as income level, water fluoridation coverage, and parental education. However, these associations should be interpreted with caution due to the ecological nature of the data and several important limitations. In particular, water fluoridation coverage showed an association with lower prevalence of dental caries in permanent teeth, though limited country representation warrants cautious interpretation. The small number of countries with high water fluoridation coverage, the use of national-level rather than individual-level indicators, and the potential for detection bias may affect the validity and generalizability of the findings. While our findings support the preventive role of water fluoridation, further longitudinal and context-specific studies are needed to elucidate causal relationships before recommending global implementation strategies, particularly as foreign aid interventions. Interestingly, middle-income countries and countries with higher average parental education levels demonstrated higher reported prevalence of dental caries. These results may reflect complex socioeconomic and behavioral dynamics and potential detection bias, rather than a direct causal relationship. In conclusion, the study contributes to the global evidence base by identifying broad socioeconomic patterns in oral health of children, and underscores the need for targeted, evidence-informed public health strategies, particularly in middle-income regions.

## Data Availability

All the data were from open available online databases.
